# PB1-F2 Proteins from H5N1 and 20^th^ Century Pandemic Influenza Viruses Cause Immunopathology

**DOI:** 10.1371/journal.ppat.1001014

**Published:** 2010-07-22

**Authors:** Julie L. McAuley, Jerry E. Chipuk, Kelli L. Boyd, Nick Van De Velde, Douglas R. Green, Jonathan A. McCullers

**Affiliations:** 1 Department of Infectious Diseases, St. Jude Children's Research Hospital, Memphis, Tennessee, United States of America; 2 Department of Immunology, St. Jude Children's Research Hospital, Memphis, Tennessee, United States of America; 3 Department of Pathology, Division of Comparative Medicine, Vanderbilt University, Nashville, Tennessee, United States of America.; National Institutes of Health, United States of America

## Abstract

With the recent emergence of a novel pandemic strain, there is presently intense interest in understanding the molecular signatures of virulence of influenza viruses. PB1-F2 proteins from epidemiologically important influenza A virus strains were studied to determine their function and contribution to virulence. Using 27-mer peptides derived from the C-terminal sequence of PB1-F2 and chimeric viruses engineered on a common background, we demonstrated that induction of cell death through PB1-F2 is dependent upon BAK/BAX mediated cytochrome *c* release from mitochondria. This function was specific for the PB1-F2 protein of A/Puerto Rico/8/34 and was not seen using PB1-F2 peptides derived from past pandemic strains. However, PB1-F2 proteins from the three pandemic strains of the 20^th^ century and a highly pathogenic strain of the H5N1 subtype were shown to enhance the lung inflammatory response resulting in increased pathology. Recently circulating seasonal influenza A strains were not capable of this pro-inflammatory function, having lost the PB1-F2 protein's immunostimulatory activity through truncation or mutation during adaptation in humans. These data suggest that the PB1-F2 protein contributes to the virulence of pandemic strains when the PB1 gene segment is recently derived from the avian reservoir.

## Author Summary

There is presently great interest in understanding how influenza viruses cause disease. In this paper, we explore the role of the influenza virus PB1-F2 protein in disease. We show that the ability of the protein to cause cell death is mediated through a mitochondrial death pathway controlled by proteins called BAX or BAK. However, this function of the protein only seems to be relevant to a restricted set of viruses and not past pandemic strains. Instead, the ability to generate inflammation in the lung proves to be a common trait of all past pandemic strains as well as the H5N1 highly pathogenic avian influenza strains which remain a significant pandemic threat. It appears likely that this pro-inflammatory phenotype is a characteristic of viruses emerging from the avian reservoir and is therefore important for new strains that cross the species barrier and establish themselves in humans. During circulation and adaptation in the mammalian lung, this function is typically lost. Of note, the novel 2009 H1N1 pandemic strain does not express a full-length PB1-F2. Were it to acquire a fully functional, inflammatory PB1-F2 through reassortment, this could herald greatly enhanced disease potential.

## Introduction

For the first time in more than 40 years, a novel influenza virus has emerged from an animal reservoir and caused a human pandemic [1]. In contrast to the three pandemics of the 20^th^ century, most disease from the novel H1N1 pandemic influenza virus has been mild [2]. The reasons for this disparity in pathogenesis are unclear because the molecular signatures of virulence of pandemic viruses are not known. A comparison of the genesis of the three 20^th^ century pandemics reveals that only 2 unique gene segments were reassorted from the avian reservoir in every case - hemagglutinin (HA) and PB1 [3]. It is accepted that antigenic novelty is required for a virus to achieve pandemic status, explaining why the HA gene has been new in past pandemics. However, the significance of inclusion of a novel PB1 gene segment in each of the 20^th^ century pandemics is not yet understood. The discovery of the PB1-F2 protein, which is translated from the +1 reading frame of the PB1 gene segment, may be a clue [4]. If this gene product contributes to virulence, it may serve as a marker of the pathogenicity of pandemic strains. Intriguingly, the 2009 pandemic H1N1 contains a PB1 gene segment that is thought to have been derived from human viruses and to have been evolving in swine for approximately 15 years [5], [6]. Its PB1-F2 open reading frame is truncated after a predicted 11 amino acids and is therefore likely non-functional if it is expressed.

PB1-F2 is a short (87–90 a.a.) influenza A virus protein discovered in 2001 [4]. After expression, it is rapidly degraded and it is not required for viral replication in ovo or in cultured cells [4]. The PB1-F2 protein is recognized by the human immune system, resulting in both humoral and T-cell responses during infections with seasonal H3N2 or highly pathogenic H5N1 viruses [4], [7], [8]. Expression of PB1-F2 has been shown to enhance viral pathogenicity in mouse models of influenza A virus infection [9], [10].

The precise contribution of PB1-F2 to virulence and its function in the life cycle of influenza A viruses in mammalian hosts are unclear. The C-terminal portion of the PB1-F2 open reading frame contains a mitochondrial targeting sequence [4]. Expression of full length PB1-F2 has been associated with mitochondrial targeting and apoptosis [4], [11] and it has been suggested that mitochondrial disruption with subsequent cell death could contribute to virulence [11], [12]. A suggested second function of the PB1-F2 protein, causing immunopathology by enhancing the inflammatory response, has been demonstrated in animal models of influenza disease [10], [13]. This mechanism for increased pathogenicity was particularly striking when the PB1-F2 from the 1918 pandemic strain was expressed, arguing for clinical relevance [10], [13]. Finally, the PB1-F2 protein has been shown to bind PB1 in vitro, and it has been proposed that this might enhance transcription by increasing nuclear retention time [14]. Downstream effects on replication were predicted to enhance virulence. We have recently demonstrated, however, that this third property of PB1-F2 is cell-type and virus strain specific and does not result in changes in viral lung load or pathogenicity in vivo [15].

Building on the results from our recent report examining the effects of PB1-F2 on viral replication [15], we analyze here the PB1-F2 protein's contribution to pathogenesis in a mouse model in the context of the other two proposed mechanisms, cell death and enhanced inflammation. To demonstrate the relevance of the results to human disease, PB1-F2 proteins from a variety of epidemiologically important influenza A virus strains including all pandemic strains from the 20^th^ century, a highly pathogenic avian influenza virus of the H5N1 subtype, and representative seasonal strains were utilized. We show here that the ability to cause cell death through PB1-F2 mediated mitochondrial interactions is specific to the laboratory strain A/Puerto Rico/8/34 (H1N1; PR8) among the viruses studied, arguing this function is not a likely contributor to pathogenicity in humans with most epidemiologically relevant strains. However, the capacity to enhance the inflammatory response is a general feature of PB1-F2 proteins encoded by PB1 genes that are direct introductions from the avian gene pool. These PB1 genes contributed to the formation of all pandemic strains of the 20^th^ century and from the currently circulating highly virulent H5N1 strain that constitutes an imminent pandemic threat. The significance of these findings for future pandemic preparation and for understanding of the current H1N1 pandemic are discussed.

## Results

### PB1-F2 induction of cell death is virus strain specific

Influenza viruses have the capability to subvert host immunity by several mechanisms. It has been suggested that down-regulation of the host immune response through apoptosis of responding immune effector cells by PB1-F2 might contribute to virulence [12]. However, effects of PB1-F2 on cell death have been contextual; the protein's apoptotic effects have been cell type specific, have differed dependent on infection with whole virus vs. expression of PB1-F2 alone, and to this point have only been studied using the laboratory strain PR8. We reasoned that if this mechanism formed an integral component in the influenza A virus strategy for down-regulation of the host immune response to infection, then induction of cell death would be mediated by PB1-F2 proteins from several epidemiologically important influenza A virus strains.

**Figure 1:**
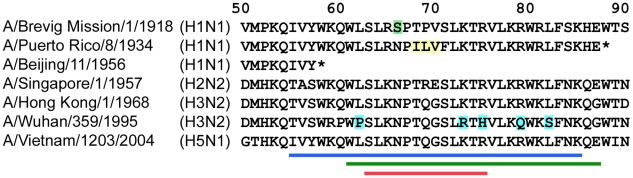
PB1-F2 sequences of viruses used in this study. A comparison of the predicted amino acid sequences of the C-terminal regions of PB1-F2 proteins of several epidemiologically important strains is presented. Colored lines at the bottom of the Figure represent the amino acid composition of the predicted helical region (blue), the 27-mer peptides used in this study (green), and the predicted mitochondrial targeting sequence (red). Blue shading highlights differences in predicted amino acid sequence between the seasonal (1995) and pandemic (1968) H3N2 strains. Yellow shading indicates amino acids predicted to be involved in the cell death phenotype of PR8 (see discussion). Green shading highlights the N66S mutation, a predicted virulence determinant for the 1918 pandemic strain [13].

Peptides derived from PB1-F2 C-terminal sequences of PR8, the three 20^th^ century pandemic strains (1918, 1957, 1968), a recent highly pathogenic avian influenza virus of the H5N1 subtype, and a recent seasonal H3N2 strain were synthesized for study [10], [15] (Figure 1). Recent seasonal H1N1 strains express a form of PB1-F2 truncated after 57 amino acids, lacking the mitochondrial targeting sequence located in the C-terminal region, and thus could not be evaluated in this manner [15], [16]. The predicted secondary structures of these peptides were determined by circular dichroism and match the reported structure of this region as described for full-length protein and 44-mer peptides [17], [18] (data not shown). These peptides are internalized when presented to cells in vitro, and are observed to have similar intracellular distributions and kinetics of degradation as does full length protein expressed from the virus (Figure S1; Text S1).

**Figure 2:**
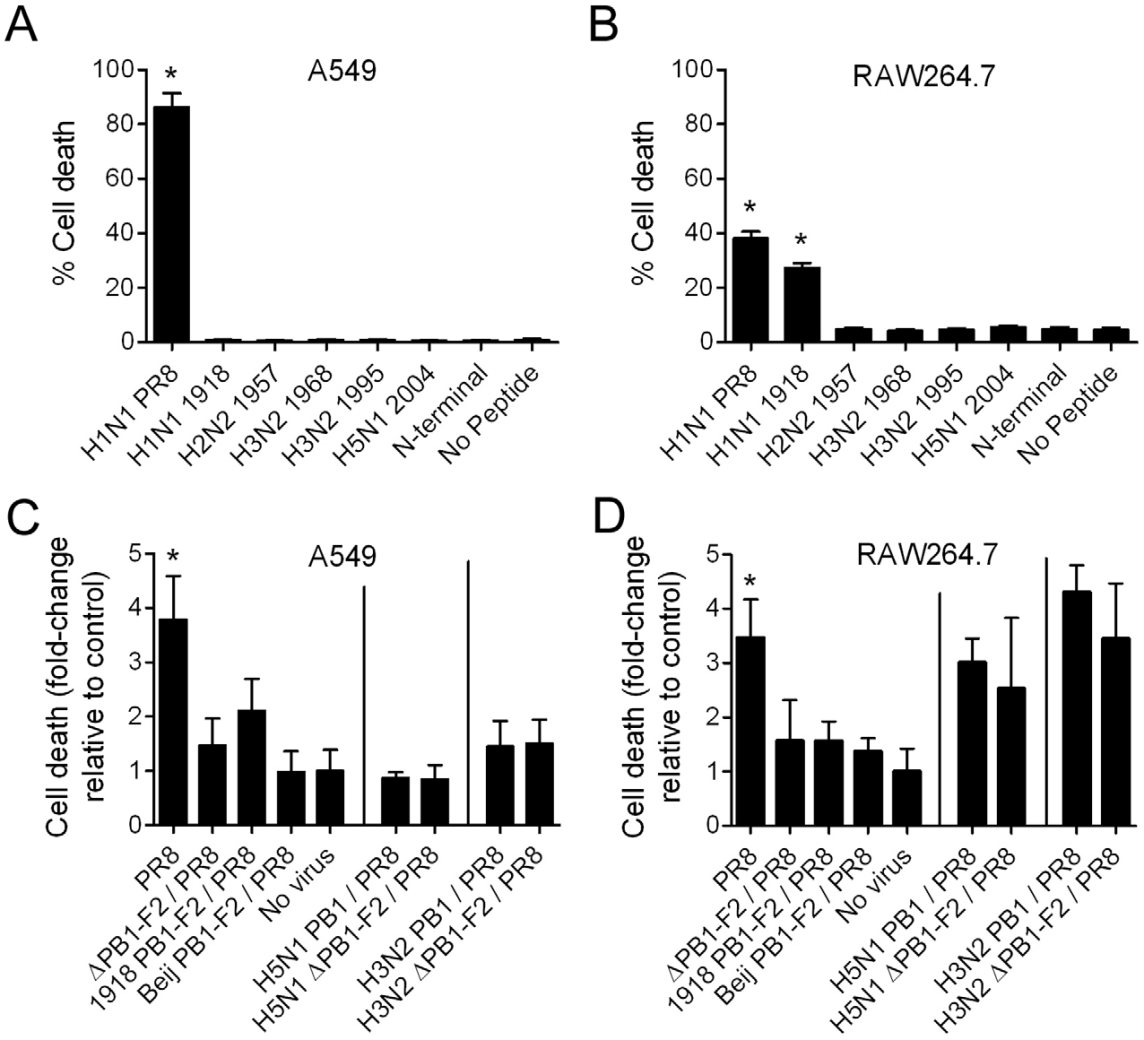
PB1-F2 causes cell death. Induction of cell death was determined in human epithelial A549 and mouse macrophage RAW264.7 cell lines by detection of Annexin^+^PI^+^ events after A & B) exposure to a panel of 27-mer peptides derived from the C-terminal region of the PB1-F2 protein (see Figure 1 for definition), an N-terminal 27-mer peptide derived from PR8 (final concentration 50 µM for each peptide), or no peptide for 1 hour; and infection with a panel of recombinant virus (see methods for definitions of viruses; the H3N2 PB1 is from A/Wuhan/359/95) at a multiplicity of infection of 1.0 at C) 12 hours and D) 8 hours post-infection. An asterisk (*) indicates a significant difference compared to A) all other peptides and controls, B) all peptides and controls except PR8 and 1918, C &D) all viruses with a PB1 gene segment derived from an H1N1 virus and the no virus control by ANOVA (p<0.05).

First, we exposed the human lung epithelial cell line A549 and the BalbcJ mouse derived macrophage cell line RAW264.7 to the panel of peptides for 1 hour at a final concentration of 50µM. Evaluation of necrosis via flow cytometric analysis of AnnexinV^+^PI^+^ events revealed that only the peptide derived from the laboratory strain PR8 induced a significant amount of cell death in the human epithelial cell line A549 (Figure 2A). Both PR8 and the peptide derived from the 1918 pandemic strain caused cell death in RAW264.7 cells (Figure 2B), but the 1918 peptide had no effect in A549. Exposure of RAW264.7 or A549 cells to peptides derived from virus strains other than those derived from PR8 or the 1918 strain did not affect viability, similar to exposure to an N-terminal peptide control or unexposed controls. There were negligible AnnexinV^+^ only events in either cell line exposed to peptides, which suggests that the cells were dying as a result of necrosis in this assay, rather than apoptosis.

To evaluate whether PB1-F2 protein expression in the context of viral infection had similar effects on induction of cell death, we next infected A549 or RAW264.7 cells with a panel of recombinant viruses at an MOI of 1 for 2–12 hours. This panel included previously described viruses unable to express PB1-F2 (ΔPB1-F2/PR8), or expressing the PB1-F2 of the 1918 pandemic strain (1918 PB1-F2/PR8) or the truncated 1956 H1N1 strain (Beij PB1-F2/PR8) [10], [15]. In addition, two previously described viruses [15] in a 7 gene segment PR8 background (7∶1 reassortants) and encoding PB1 gene segments from a 2004 highly pathogenic avian influenza of the H5N1 subtype (H5N1 PB1/PR8) or from a 1995 human H3N2 strain (H3N2 PB1/PR8) were utilized along with their isogenic deletion mutants for PB1-F2 (H5N1 ΔPB1-F2/PR8 and H3N2 ΔPB1-F2/PR8). Using confocal microscopy we demonstrated that PB1-F2 proteins from the viruses which were utilized in this study and which have an intact PB1-F2 ORF are expressed and distribute throughout the cell (Figure S2; Text S2) and [15].

As has been demonstrated previously [4], [11], [12], PR8 virus induces significant cell death compared to uninfected controls (Figure 2C, D). PB1-F2 has been shown to be maximally produced at 6–8 hours post-infection in these cells types [15] and only minimal cell death was appreciated in the first 6 hours post infection (data not shown). When RAW264.7 cells were infected with PR8 virus, necrotic death peaked 8 hours after infection, while in A549 cells it peaked 12 hours post infection. The inability of the ΔPB1-F2/PR8 to express PB1-F2 and the truncation of the C-terminal portion of Beij PB1-F2/PR8 abrogated the ability of both viruses to cause cell death in both cell types (Figure 2C, D). Interestingly, however, expression of the 1918 PB1-F2, which has been associated with enhanced virulence [10], [13], did not cause significant increases in cell death over controls. In addition, deletion of PB1-F2 in either an H3N2 or H5N1 PB1 gene segment background did not alter the cell death phenotype of chimeric viruses expressing those PB1s. We conclude from these data that the ability to cause cell death through PB1-F2 is a property of specific strains and is unlikely to have contributed to the pathogenicity of the 20^th^ century influenza virus pandemics or recent seasonal epidemics.

### PR8 PB1-F2 promotes BAK/BAX-dependent mitochondrial outer membrane permeabilization

It has been suggested that PB1-F2 is able to induce cell death by disrupting mitochondrial organization through interaction with inner mitochondrial membrane adenine nucleotide translocator 3 (ANT3) and the outer mitochondrial membrane voltage channel 1 (VDAC1) to produce a mitochondrial permeability transition [12]. Alternatively, the PR8 PB1-F2 has been shown to permeabilize planar membranes, and thus may be able to directly permeabilize mitochondria [19]. We therefore probed the mechanism underlying the induction of cell death mediated by the PR8 PB1-F2. We hypothesized that the PB1-F2 C-terminal domain may promote mitochondrial outer membrane permeabilization (MOMP) via the activation of the pro-apoptotic Bcl-2 family effector proteins BAX and BAK.

**Figure 3:**
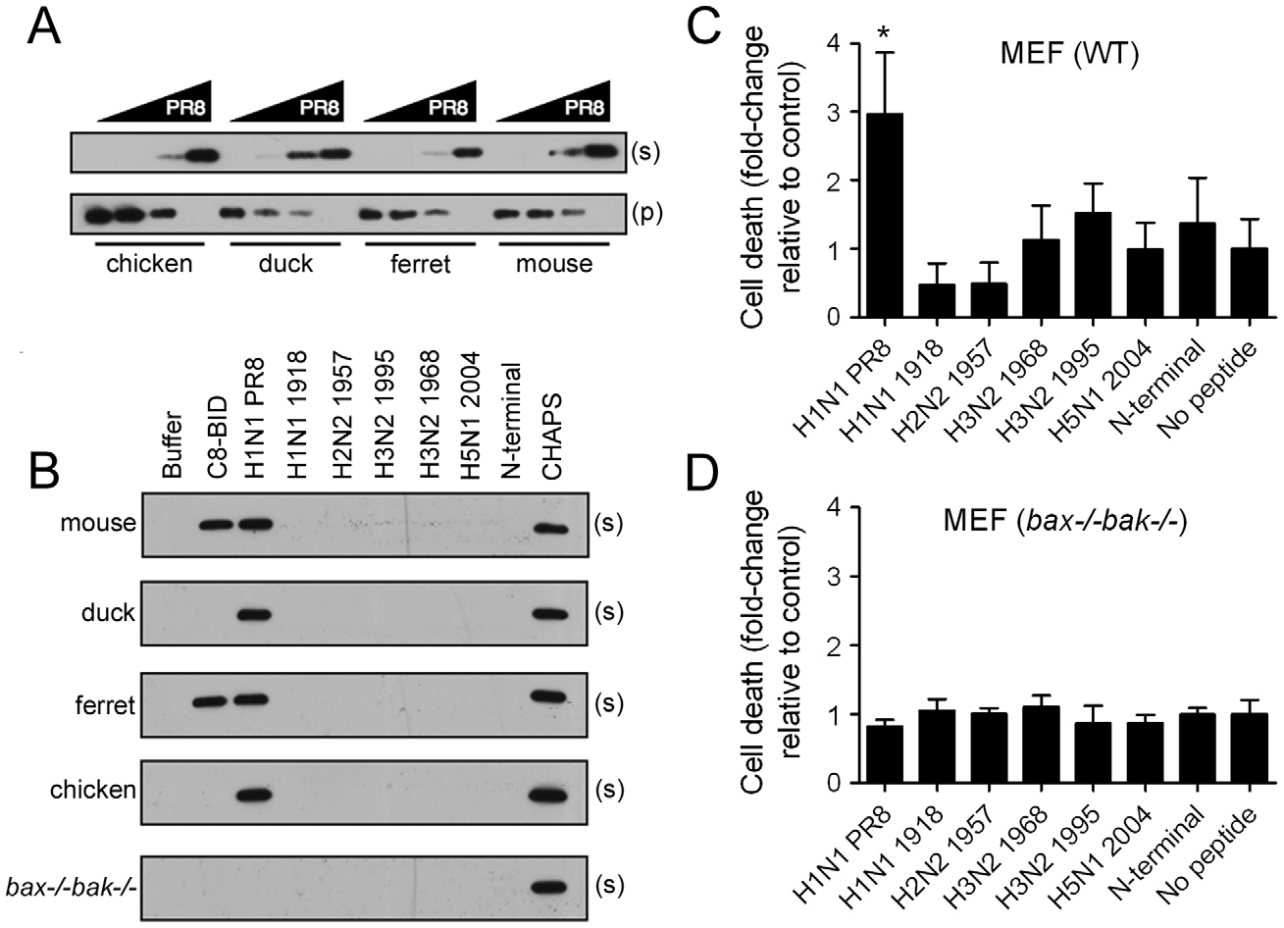
PB1-F2 mediated cell death is virus strain specific. A & B) Analysis of MOMP via detection of cytochrome *c* release by western blot after exposure to a panel of peptides (see Figure 1 for definitions) of heavy membrane fractions (mitochondria) derived from chicken, duck, ferret and mouse livers (B includes mitochondria from *bax-/-bak-/-* mice). A) Mitochondria exposed to 1, 10, 100 and 1000 µM PR8 peptide for 1 hour show release of cytochrome *c* into the supernatant (s). Pellet (p) contains intact mitochondria containing unreleased cytochrome *c*. B) Mitochondria were exposed to the peptide panel and supernatants were analyzed for cytochrome *c* release. C8-BID was used as a positive control for mammalian mitochondria. CHAPS solubilizes mitochondrial membranes, enabling release of total cytochrome *c*. C&D) Induction of cell death in C) wild-type and D) *bax-/-bak-/-* murine embryonic fibroblasts (MEFs) after a 1 hour exposure to the panel of peptides (50 µM final concentration). An asterisk (*) indicates a significant difference compared to all other peptides and controls by ANOVA (p<0.05).

To test this hypothesis, we exposed isolated mitochondria obtained from the livers of BALBcJ mice to various concentrations of PR8 derived PB1-F2 peptide. Analysis of cytochrome *c* release revealed that peptide derived from the laboratory strain PR8 could promote MOMP. As PR8 is a mouse adapted laboratory strain and initial experiments utilized only murine derived intact mitochondria, we repeated peptide exposure experiments using mitochondria obtained from liver tissue of chicken embryos, outbred ferrets, and mallards. Results indicate that PR8 PB1-F2 derived peptide is a potent inducer of cytochrome *c* release and is not species specific (Figure 3A). As little as 10µM PR8 PB1-F2 peptide was sufficient for inducing cytochrome *c* release from each species analyzed. As different PB1-F2 proteins may display species specificity in mitochondrial targeting by the PB1-F2 protein, we next examined MOMP and release of cytochrome *c* using the panel of different C-terminal PB1-F2 peptides and an N-terminal peptide from PR8 as a negative control. Again, only the PR8 PB1-F2 was able to promote MOMP (Figure 3B), irrespective of the species from which the mitochondria were obtained. In contrast, mitochondria obtained from the livers of *bak-/-bax-/-* mice did not release cytochrome *c* when incubated with PB1-F2 C-terminal peptide (Figure 3B). These data suggest that apoptosis induced by the PR8 PB1-F2 protein is dependent upon BAK/BAX activation and MOMP.

To determine BAK/BAX dependency for PR8 PB1-F2 induction of apoptosis at the cellular level, we treated wild-type murine embryonic fibroblasts (MEFs) and *bak-/-bax-/-* MEFs with the panel of PB1-F2 peptides and analyzed for apoptosis. The PR8 PB1-F2 derived peptide induced apoptosis in wild-type MEFs, but peptides derived from other viruses did not show similar activity (Figure 3C). However, apoptosis in *bak-/-bax-/-* MEFs was greatly reduced such that PR8 PB1-F2 induction of cell death was equivalent to cells exposed to the other peptides (Figure 3D). Taken together, the cytochrome *c* release and apoptosis data implicate PR8 PB1-F2 in BAK/BAX activation and the mitochondrial pathway of apoptosis, but suggest that this effect is specific to certain influenza virus strains.

### PB1-F2 causes increased cellularity in bronchoalveolar lavage fluid

PB1-F2 proteins from both laboratory viruses and clinically relevant strains have been shown to contribute to primary viral virulence [9], [10], [13] and to the pathogenesis of secondary bacterial infections [10] in mice. This appears not to be related to the proposed ability of PB1-F2 to enhance replication or cause cell death, as both of these functions are limited to specific virus strains ([15] and this report). We therefore investigated the contribution of several clinically relevant PB1-F2 proteins to immunopathology.

**Figure 4:**
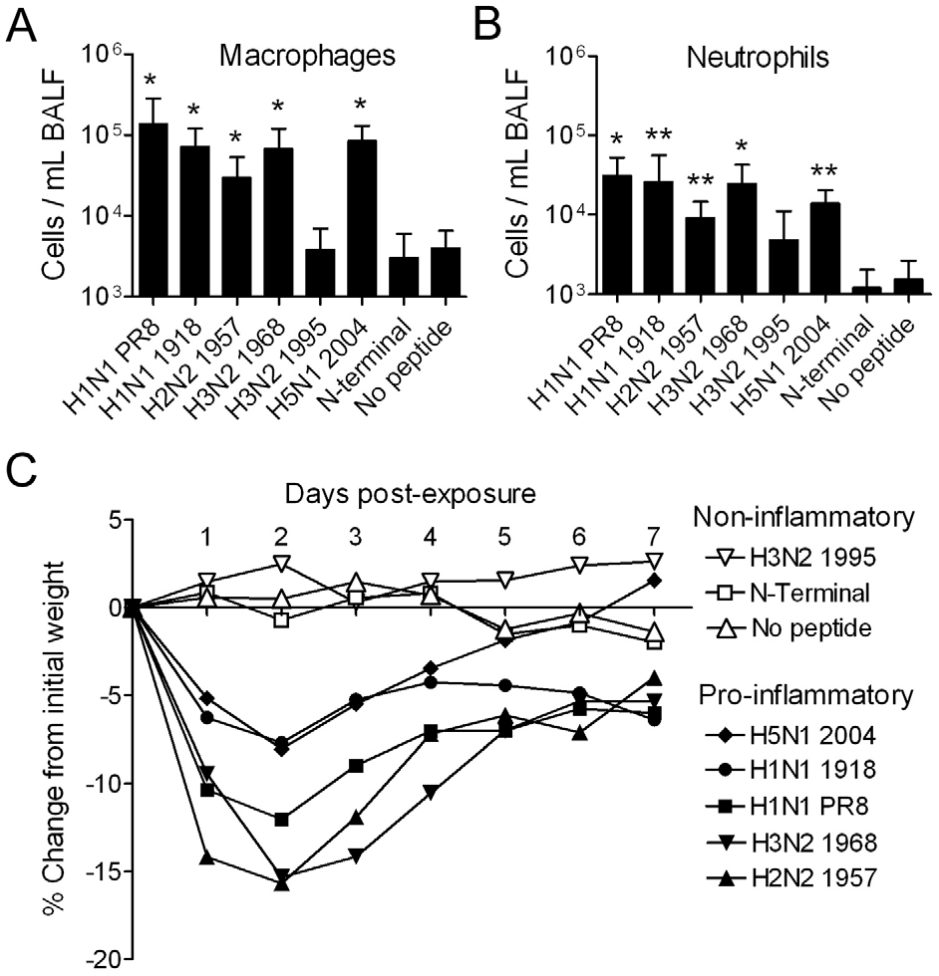
PB1-F2 peptides enhance inflammation in the lung. Groups of mice were exposed to a panel of peptides (100 mM final concentration, see Figure 1 for definitions) and euthanized 24 hours later. BAL fluid from the animals was assayed by flow-cytometry [10] for the mean number of A) macrophages and B) neutrophils compared to unexposed mice or mice exposed to an N-terminal control peptide. An asterisk (*) indicates a significant difference compared to the 1995 H3N2 peptide and controls by ANOVA (p<0.05). A double asterisk (**) indicates a significant difference compared to the controls. Error bars indicate the standard deviation from the mean. C) In a separate set of experiments, mice exposed to peptide were monitored for overt signs of illness and percentage change from initial weight. For clarity, peptides are grouped by their inflammatory potential as determined in A & B.

Mice were exposed to a panel of PB1-F2 derived peptides and were euthanized 24 hours later for collection of bronchoalveolar lavage fluid (BALF). C-terminal PB1-F2 peptides derived from PR8, the pandemic strains from 1918 (H1N1), 1957 (H2N2) and 1968 (H3N2), and the 2004 H5N1 virus all caused significant influx of white blood cells into the BALF compared to controls. Several cell types were increased including T-cells (data not shown), dendritic cells (data not shown), macrophages (Figure 4A) and neutrophils (Figure 4B). Interestingly, the peptide derived from a more recent H3N2 strain, A/Wuhan/359/1995, did not cause an appreciable increase in inflammatory cells over controls. When peptide exposed mice were followed for morbidity for 7 days, striking differences were seen comparing the five peptides that caused inflammation with the 1995 H3N2 peptide and the controls. The “pro-inflammatory” peptides caused huddling, hunching, labored breathing, ruffled fur and weight loss which peaked in the first 24–48 hours after exposure, while the “non-inflammatory” exposures caused no clinical signs (data not shown) or weight loss (Figure 4C). Thus, the ability to cause lung inflammation appears to be a property of PB1-F2 proteins recently emerged from the avian gene pool. H1N1 strains circulating in humans since about 1950 have a truncated PB1-F2 that lacks the C-terminal residues responsible for this effect and in contrast to their pandemic forbear from 1968, recently circulating H3N2 strains lack a PB1-F2 capable of causing inflammation.

**Figure 5:**
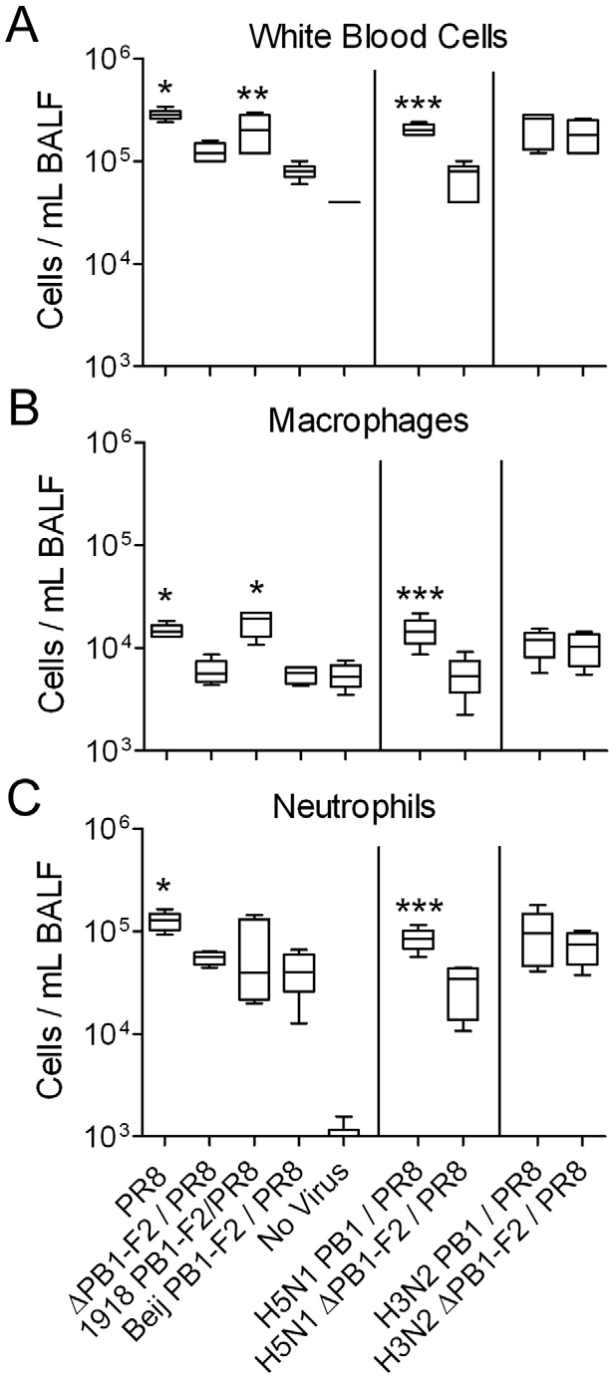
PB1-F2 enhances viral-mediated lung inflammation. Groups of mice were infected with a panel of viruses (see methods for definitions) and euthanized 72 hours post-infection. BAL fluid from the animals was assayed by flow-cytometry [10] for the mean number of A) white blood cells, B) macrophages, and C) neutrophils compared to uninfected mice or mice infected with a virus lacking the ability to express PB1-F2. An asterisk (*) indicates a significant difference compared to animals infected with the ΔPB1-F2/PR8 or Beij PB1-F2/PR8 viruses, and uninfected control animals by ANOVA (p<0.05). A double asterisk (**) indicates a significant difference compared to animals infected with the Beij PB1-F2/PR8 virus and uninfected control animals. A triple asterisk (***) indicates a significant difference compared to animals infected with the H5N1 ΔPB1-F2/PR8 virus and uninfected control animals. Error bars indicate the standard deviation from the mean.

To assess the contribution of PB1-F2 to inflammation in the context of a full virus, where expression of other proteins is likely to contribute to the inflammatory response, we utilized the previously described set of viruses on the PR8 backbone in a mouse infection model. In data previously reported with these viruses, alteration or abrogation of PB1-F2 did not result in significant differences in weight loss, in the dose needed to cause death in mice, or in viral lung titers 1, 3, 5 and 7 days after infection for any comparisons except those previously reported for the 1918 PB1-F2 [10], [15]. However, the differential effects of PB1-F2 expression could clearly be seen in the inflammatory response in the lungs. Disruption of PB1-F2 expression in PR8 or replacement of the PR8 PB1-F2 with the C-terminally truncated Beij PB1-F2 both significantly reduced the number of white blood cells found in BALF 3 days post viral infection (Figure 5A) compared to PR8. This significant difference was maintained when specifically assessing the influx of either macrophages or neutrophils (Figure 5B, C). Expression of the 1918 PB1-F2 led to effects similar to those of the PR8 virus. Use of the virus containing the H5N1 PB1 gene segment in a PR8 background revealed that disruption of PB1-F2 expression also significantly depressed the inflammatory response compared to the virus maintaining the ability to express full length PB1-F2 (Figure 5A, B, C). However, in agreement with the peptide data, no differences were seen that could be attributed to the 1995 H3N2 derived PB1-F2.

### PB1-F2 causes inflammatory changes in the lung

**Figure 6:**
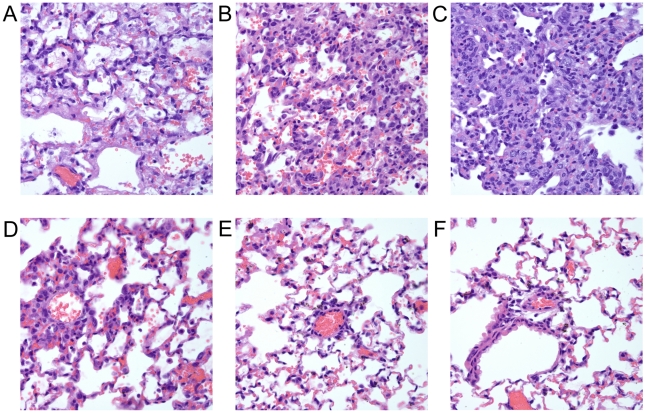
PB1-F2 derived peptides cause inflammation and lung pathology. Groups of mice were exposed to a panel of peptides (100 mM final concentration, see Figure 1 for definitions) and euthanized 72 hours later. Lungs were removed, sufflated, sectioned, and stained with hematoxylin and eosin for histopathologic evaluation. Representative 40× sections are shown from mice exposed to peptides derived from the PB1-F2 protein of A) PR8, B) H1N1 1918, C) H2N2 1957, D) H5N1 2004, E) H3N2 1995, or F) no peptide control.

Having determined that PB1-F2 drives an influx of inflammatory cells into the lungs that is detectable in BAL fluid, we next assessed the impact of this enhanced inflammatory response on histopathologic changes in lung tissue. Groups of mice were exposed to the panel of peptides and euthanized 24 or 72 hours later for examination of the lungs by an experienced veterinary pathologist (K.L.B.) who was blinded to the purpose of the study and the composition of the groups. At the 24 hour timepoint, only minimal perivascular changes were observed and no differences were apparent between groups (data not shown). 72 hours after exposure, however, significant pathology was observed in some groups of mice compared to others, mirroring the dichotomy in morbidity pictured in Figure 4C. Only minimal rare, perivascular infiltration of lymphocytes around small vessels was observed in mice exposed to the N-terminal control peptide or the peptide derived from the H3N2 seasonal strain A/Wuhan/359/1995 (Figure 6E, F). However, in mice exposed to peptides derived from the pandemic strains from 1918 and 1957, as well as the peptide from a 2004 H5N1 strain, hypertrophy of type II pneumocytes and thickening of alveolar septae were observed. Significant infiltration of neutrophils and macrophages was noted in both interstitial perivascular regions as well as within alveolar spaces (Figure 6B–D). Macrophages grossly outnumbered neutrophils, mirroring the findings in BAL fluid following peptide exposure (Figure 4A, B).

In the lungs of mice exposed to the PR8 peptide, similar inflammatory changes were seen, but there was an additional finding of direct alveolar wall damage with fibrin deposition and copious necrotic debris filling the alveoli, suggestive of cell death (Figure 6A). From these data and data presented in Figure 4, we conclude that the major effect of exposure of mammalian lungs to PB1-F2 proteins is generation of a macrophage and neutrophil dominated inflammatory response which causes significant immunopathology. Following adaptation of an H3N2 to humans, this function has been lost. The additional capability of the PR8 PB1-F2 protein to cause cell death enhances the pathology seen in the animals resulting in worse clinical outcomes. In titration of this panel of peptides in mice, only the PR8 peptide caused fatal illness (data not shown), suggesting that both functions of PB1-F2 can contribute to fatal viral pneumonia.

**Figure 7:**
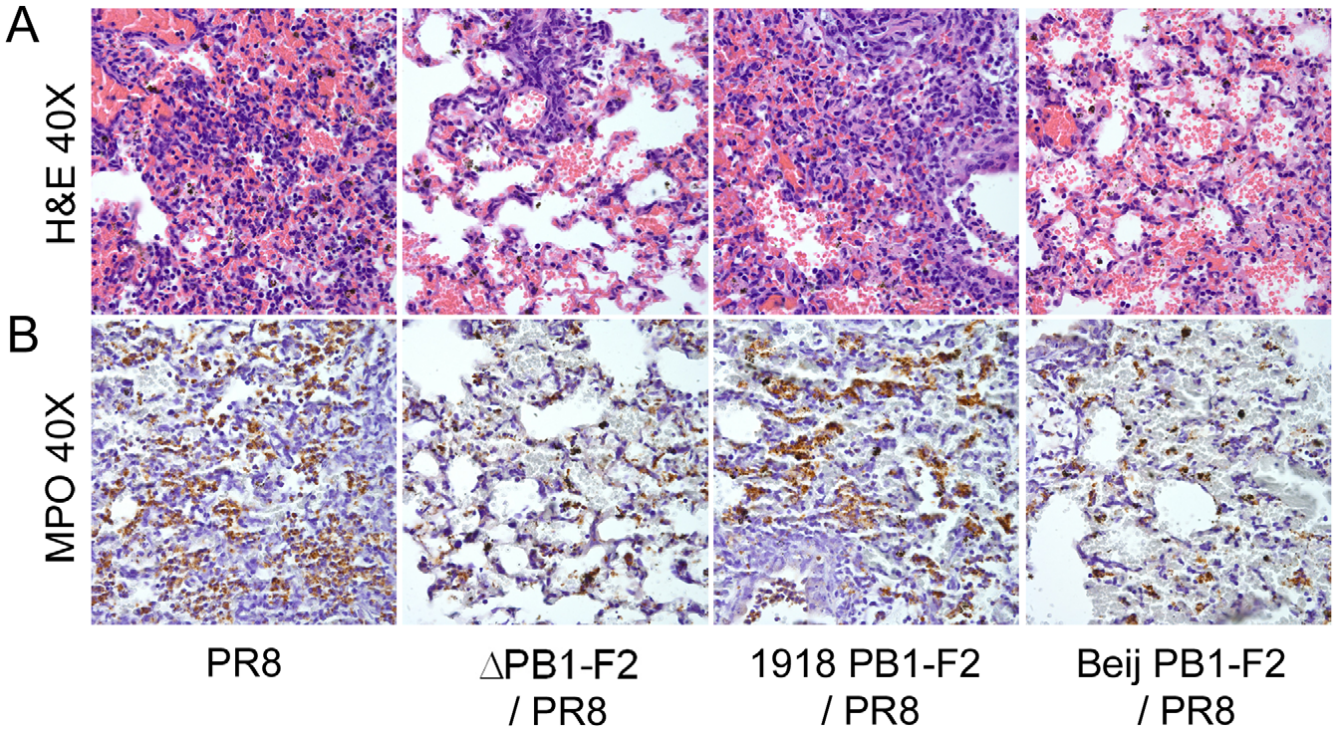
PB1-F2 contributes to influenza virus mediated immunopathology. Groups of mice were infected with a panel of viruses as indicated (see methods for definitions) and euthanized 72 hours post-infection. Lungs were removed, sufflated, sectioned, and examined for histopathology following A) staining with hematoxylin and eosin or B) application of an antibody specific for MPO followed by a horse-radish peroxidase-conjugated secondary antibody, staining with chromagen DAB, and counterstaining with hematoxylin.

To assess this inflammatory phenotype in the context of a full virus, mice were infected with WT PR8, 1918 PB1-F2/PR8, ΔPB1-F2/PR8, or Beij PB1-F2/PR8 and were euthanized 72 hours later for histopathologic examination as described above. In all lungs examined, pathologic changes typical of PR8 viral infection were observed, including perivascular infiltration of lymphocytes into interstitial regions, areas of focal necrosis of terminal airways and prominent cellular debris associated with acute hemorrhage into alveoli (Figure 7A). In the lungs of mice infected with PR8 or 1918 PB1-F2/PR8, however, significantly more perivascular cuffing was noted, consisting of an admixture of both viable and degenerate neutrophils and macrophages. The number of inflammatory cells seen in both the interstitium and alveoli was noticeably greater in these mice than in mice infected with ΔPB1-F2/PR8 or Beij PB1-F2/PR8. This finding supports the BAL data presented in Figure 5 and the histopathologic changes caused by the PB1-F2 peptide alone in Figure 6. Staining for myeloperoxidase (MPO) highlighted the influx of granulocytes and resulting inflammation in the lungs of mice infected with PR8 or 1918 PB1-F2/PR8 and was significantly more intense in these lungs compared to the ΔPB1-F2/PR8 or Beij PB1-F2/PR8 infected lungs (Figure 7B). We conclude that expression of full length PB1-F2 enhances lung inflammation during influenza virus pneumonia and increases the pathologic damage that occurs. During infections with viruses such as PR8 that are capable of causing PB1-F2 mediated cell death and inflammation by an unrelated mechanism, the lung injury is enhanced.

## Discussion

The data presented here demonstrate the capacity of the influenza A virus PB1-F2 protein to enhance the lung inflammatory response in a mouse model. This study is the first to explore the function of PB1-F2 utilizing PB1-F2 proteins from several different, epidemiologically important influenza A virus strains. These strains included those containing novel PB1 gene segments contributing to the formation of all pandemic strains of the 20th century and from the currently circulating, highly virulent H5N1 strains that constitute an imminent pandemic threat. The inflammatory response engendered by PB1-F2 was characterized by increased cellular infiltrates into the interstitial and alveolar spaces of the lungs, which largely consisted of macrophages and neutrophils. Among the viruses assessed, induction of inflammation by PB1-F2 was a property restricted to proteins encoded by PB1 gene segments that were direct introductions from the avian gene pool. Seasonal strains which had adapted to humans lacked a PB1-F2 capable of causing inflammation. One important caveat is that these experiments were done on a PR8 background, so further study of these PB1-F2 proteins in their native background is indicated. Our analysis of MOMP utilizing mitochondria from several animal species and study of cell death relating to PB1-F2 expression implicate the PR8 PB1-F2 in BAK/BAX activation and the mitochondrial pathway of apoptosis. However, induction of cell death via mitochondrial association was specific only to the PB1-F2 protein of the laboratory strain of PR8 in these studies and is not a general property of all PB1-F2 proteins.

All influenza viruses infecting humans are zoonotic reassortants comprised of gene segments that derive ultimately from an avian reservoir of precursor strains. It has been appreciated for some time that one of the requirements for emergence of a pandemic strain is antigenic novelty of the HA, which facilitates high clinical attack rates [20]. However, the molecular signatures contributing to another criterion for pandemic strains, the ability to cause serious disease, are not clear at present. The inclusion of a novel PB1 gene segment in each of the virulent 20^th^ century pandemics may be an intriguing clue. Evidence presented here implicates full length PB1-F2 protein expression, encoded by a +1 open reading frame of the PB1 gene segment, as a marker of pathogenicity of 20^th^ century pandemic strains. The pro-inflammatory capability of the H5N1 PB1-F2 demonstrated by this study provides further reason for concern that this subtype represents a serious pandemic threat, although significant sequence diversity exists among avian strains and the specific molecular signatures for the inflammatory phenotype are not yet known. Full length PB1-F2 proteins are expressed by nearly all avian influenza virus strains [16], but analysis of lineages after introduction of these strains into humans or pigs show evidence of permanent truncation during adaptation in mammalian hosts. This pattern implies that there is some functional utility for the protein in birds that is not evolutionarily advantageous for the virus in mammals. We speculate that the inflammatory property of PB1-F2 described here facilitates transmission from the natural host niche of influenza viruses in waterfowl, the gut, by increasing luminal secretions. If induction of inflammation in the lung is detrimental or neutral to the virus during its life-cycle in mammalian hosts, this function might be lost over time during adaptation despite a lack of evidence for strong positive selection pressure on the protein [21]. This is an obvious area for further study in relevant species.

In humans, the H1N1 PB1-F2 lineage introduced in 1918 became truncated around 1948–1950 (Figure 1), losing the C-terminal region that contains the mitochondrial targeting sequence [4], [22] and promotes inflammation (this report). The currently circulating H3N2 lineage PB1-F2 proteins are still full-length, but have undergone evolutionary changes during adaptation. In this study we demonstrate that PB1-F2 derived from the 1968 pandemic strain can cause lung inflammation, but that from a recently circulating seasonal H3N2 cannot. Analysis of the PB1-F2 C-terminal regions of these strains shows that only 5 amino acid differences are found within the region selected for generating the peptides used to study inflammation (Figure 1). Two of these changes, K73R and R75H, are within the MTS at positions shown by alanine substitution to be important for mitochondrial targeting [22]. However, both changes are conservative in terms of charge, and the A/Wuhan/359/1995 PB1-F2 shows mitochondrial localization by confocal microscopy (Figure S2). This suggests alteration of mitochondrial targeting by mutation is not responsible for the change in phenotype. A clear priority is to understand which of these mutations, or what combination of them, is responsible for the diminished capacity of the 1995 H3N2 strain to generate inflammation. This may allow prediction of which avian or swine PB1 gene segment precursors represent a pandemic threat were they to reassort onto a virus crossing the species barrier. Only one other amino acid change, the N66S change in the 1918 PB1-F2 (Figure 1), has been associated with a gain in virulence [13]. However, an S at position 66 is not necessary for enhancement of inflammatory responses since the other pro-inflammatory PB1-F2 proteins studied here do not possess this residue at that position.

One of the hypotheses advanced in our earlier work [10] to explain the immunopathogenic phenotype of the 1918 pandemic strain PB1-F2 was that the previously described cell death phenotype [4], [11] was responsible for the inflammatory response by killing lung cells resulting in activation of local host defenses. However, the dissociation between cell death and immunopathology demonstrated in this report, in particular the inability of the 1918 PB1-F2 to activate mitochondrial cell death pathways, suggests that the immunostimulatory activity of PB1-F2 is a direct effect. PB1-F2 is expressed early in the course of infection [10] and has a short half-life [4]. This suggests that the trigger for these inflammatory responses is likely to take place early during infection, perhaps by direct recognition of PB1-F2 by pattern recognition receptors. Finding the potential binding partner of PB1-F2 in this pathway is of obvious interest and is a focus of ongoing studies. Although antibody is generated in humans which can recognize PB1-F2 [7], it is not known whether this can modulate the functional outcomes of PB1-F2 expression and decrease disease. One additional caveat for these studies is that deletion of the PB1-F2 start codon has been shown to affect expression levels of a truncated version of PB1, the N40 protein [23]. Although N40 over-expression was not shown to alter viral replication in that study, it could have unknown effects in vivo that could complicate interpretation of our experiments with PB1-F2 knock-out viruses.

Since its discovery the consensus has been that the PB1-F2 proteins from selected viruses are pro-apoptotic, targeting the mitochondria and inducing pore formation through association with VDAC1 and ANT3 [4], [11], [12], [19], [24]. ANT3 and VDAC1 molecules are major components of the permeability transition pore of the mitochondrial membrane and enable cytochrome *c* release from the mitochondria, leading to the activation of the caspase pathway and subsequent cell death. This ionic channel however, becomes activated and induces membrane permeability, following marked oxidative stress and altered mitochondrial calcium levels [25]. Most of the available data surrounding the apoptotic pathway suggest that BAK/BAX mediation of cytochrome *c* release (irrespective of whether components of the permeability transition pore complex are involved) mark apoptotic cell death, whereas cytochrome *c* release exclusively mediated by VDAC and ANT implies non-apoptotic, mainly necrotic cell death [26]. We show conclusively that, among the viruses tested, only the PB1-F2 C-terminal domain of the PR8 laboratory strain efficiently engages the BAK/BAX pathway. The dependency for presence of BAK/BAX expression on the PR8 induced pore formation in mitochondria defines the role for PR8 PB1-F2 as a BH3 only protein that activates conformational change in the BAX complex within the mitochondria. Whether this complex leads to association with the VDAC/ANT ion channel formation, or activated BAX complex solely causes pore formation and subsequent cytochrome *c* release, is currently not understood.

Analysis of the 5944 predicted amino acid sequences of PB1-F2 proteins available in the Influenza Research Database [27] in the context of these data suggests that the ILV motif from amino acids 68–70 of the PR8 PB1-F2 ORF is likely to be involved in the cell death phenotype (Figure 1). This is the only area of the C-terminal portion of the PR8 sequence that differs between PR8 and other strains studied herein. The 68I and 69L are present in only 17 of these 5944 sequences, all from human viruses. The 70V is invariably present when the 68I and 69L are present. Fourteen of these viruses circulated between 1933 and 1947 and were of the H1N1 subtype, and only 2 viruses with published PB1-F2 sequences from this era do not share this motif. The other 3 viruses with this motif are A/Victoria/68 (H3N2), A/TW/3355/97 (H1N1), and A/Russia:St.Petersburg/8/06 (H1N1). The 1918 pandemic strain has the sequence TPV from amino acids 68–70; this motif is shared with only 8 other sequences available in the database. Having this unusual motif at this position and sharing the 70V substitution with PR8 might account for the limited ability of the 1918 PB1-F2 to cause necrosis in vitro (Figure 2), although further study is needed to confirm this hypothesis. The other viruses studied in this report have more common motifs at these three positions, and both F and S are commonly found at position 71 making it an unlikely candidate. Fine mapping of the specific amino acids required to cause the cell death phenotype is required to better determine which viruses might utilize this mechanism, whether this accounts for the limited ability of the 1918 PB1-F2 to also cause some cell death, and how likely reversion to this phenotype by mutation to the necessary sequence might be. However, the current data suggest it is a rare trait in modern seasonal viruses and in the animal reservoir.

The emergence of the pandemic H1N1 strain in 2009 has increased the urgency to understand the molecular basis of virulence of influenza viruses. The currently circulating pandemic strain does not encode a full-length PB1-F2 protein, so would not be expected to cause lung inflammation as effectively as previous pandemic strains. In addition, since the PB1-F2 has been shown to be a major virulence factor in the genesis and severity of secondary bacterial infections [10], we and others have predicted that bacterial pneumonia would be a less frequent complication during this pandemic than was seen in the 20^th^ century pandemics [28]. Indeed, although bacterial super-infections complicated more than 95% of all deaths during the 1918 pandemic [29], bacterial pneumonia has been found on autopsy in only 29% of fatalities that have been assessed during the current pandemic thus far [30]. This could change if the pandemic H1N1 were to acquire a full length PB1-F2 through reversion of the two stop codons in the PB1-F2 ORF or by reassortment with a virus encoding a full-length PB1-F2. Were reversion to occur, the full length PB1-F2 of the pandemic H1N1 more closely resembles the 1995 H3N2 strain than the 1968 pandemic precursor, having predicted residues 73R, 75H and 79Q (Figure 1). It also lacks the PR8 specific sequences seen at amino acids 68–70. Based on this analysis and the data presented in this study, it is unlikely that reversion of the stop codons or reassortment of the PB1 gene segment with currently circulating seasonal influenza strains would provide the pandemic virus with a functional, pro-inflammatory PB1-F2. Indeed, data published while this manuscript was in revision indicate that genetic reversion of the stop codons of the pandemic H1N1 strains to produce full length PB1-F2 has only minimal effects on virulence and support for secondary bacterial infections [31]. However, the less likely scenario of reassortment with an avian or swine virus with a fully functional PB1-F2 remains possible and could herald enhanced virulence and increased incidence and severity of secondary bacterial infections.

## Materials and Methods

### Cell lines and viral infections

Madin-Darby canine kidney (MDCK) cells were grown in Minimal Essential Medium (MEM) supplemented with 10% heat inactivated fetal bovine serum (FBS), 2mM glutamine and antibiotics. A549 cells were grown in F12K medium supplemented with 10% FBS, 2mM glutamine and antibiotics. Immortalized MEFS were produced by transfecting primary, unpassaged MEFs with SV40 genomic DNA using Lipofectamine 2000 (standard conditions, Invitrogen) and selected by colony formation and growth. SV40 immortalized wild type and *bak^−/−^bax^−/−^*. 293T, MEF and BAX^−/−^BAK^−/−^MEF cells were grown in DMEM medium supplemented with 10% FBS and antibiotics. Cell cultures were maintained at 37°C in a 5% CO_2_ incubator. Cells were washed once with phosphate buffered saline (PBS), infected with the amount of virus indicated by indicated multiplicity of infection (MOI) and further incubated as described previously [10].

### Generation of plasmids and viruses

A set of plasmids were generated on the pHW2000 backbone as described [15], [32], encoding for the PB1 gene segment of PR8, A/Vietnam/1203/04 (H5N1) or A/Wuhan/359/95 (H3N2). In each of these backgrounds, the open reading frame for PB1-F2 was disrupted by altering the start codon (T120C mutation by PB1 numbering) so translation will not initiate and inserting a stop codon after 11 residues (C153G) to insure a complete 1 knock-out [9], [10]. The PR8 PB1-F2 sequence was further altered by QuikChange site-directed mutagenesis (Stratagene) as described [10], [15] so that the protein expressed was identical to either the A/Brevig Mission/1/18 (H1N1; 1918 PB1-F2) or A/Beijing/11/56 (H1N1; Beij PB1-F2) virus. In no case did these mutations in the PB1-F2 reading frame cause non-synonymous mutations in the PB1 reading frame. The N40 start codon [23] was intact in all plasmids. These eight PB1 plasmids were then incorporated into a corresponding set of eight viruses on a 7 gene PR8 backbone (7∶1) by reverse genetics as described [15]. Resulting viruses were rescued by one passage in MDCK cells then propagated a single time in eggs for stocks to be used in these studies. All viruses were fully sequenced to insure no inadvertent mutations occurred during virus rescue and propagation, then characterized in tissue culture and eggs as previously described [33]. Expression or lack of expression of PB1-F2 protein was confirmed for all viruses through use of confocal microscopy (Figure S2).

### Peptide generation

Using the predicted amino acid sequences of the PB1-F2 proteins from PR8, A/Brevig Mission/1/1918, 1/Singapore/1/1957, A/Hong Kong/1/1968, A/Wuhan/359/1995 and A/Vietnam/1203/2004, peptides from the C-terminal end were synthesized as described [10]. The region covered began at amino acid 61 and extended to the termination of the protein (by PR8 sequence) at amino acid 87 (Figure 1). An additional N-terminal peptide was synthesized from the PR8 sequence as a positive control (MGQEQDTPWILSTGHISTQK) as described [10]. Peptides were either synthesized on an Apex 396 multiple organic synthesizer (Aaaptec, Luisville, KY) or purchased from GenScript Corporation (Piscataway, NJ). Immediately prior to use in animals or cell culture, peptides were suspended in PBS at a concentration of 1mM.

### Cell death assays

Peptide exposure assays were conducted in 96 well round bottom tissue culture dishes, whilst virus infection assays were conducted in 24 well plates. Cells were seeded at a density of 1×10^6^ cells/mL for 30min (peptide assays) or overnight (virus assays) in infection media (cell growth media with BSA substituted for FCS). Cells were then exposed to 50uM (final concentration) of peptide for 1h, or infected with virus at the MOI indicated for periods ranging from 2–12h. Cells from the supernatant and monolayers were then harvested, washed and stained with APC labeled Annexin and Propidium Iodide (PI) (Becton Dickinson, San Jose, CA) for 20min. After a final wash step, cells were resuspended in 100uL FACs wash buffer (PBS containing 3% BSA and 0.01% sodium azide) and analysed on the FACs Calibur (BD Biosciences) and BD CellQuest Pro software (version 5.2.1, BD Biosciences). Necrotic cellular events were defined as Annexin-V^+^ and PI^+^, whilst apoptotic events were Annexin-V^+^ only. Viable cells were considered as neither Annexin-V nor PI positive.

### Mitochondrial isolation and cytochrome c release

Heavy membrane fractions (referred to as mitochondria) were purified from 10 grams of fresh liver (duck, ferret and mouse) or 20 chicken embryos day 14, using dounce homogenization and differential centrifugation in trehalose mitochondrial isolation buffer (TMIB: 300 mM trehalose, 10 mM HEPES-KOH pH 7.4, 10 mM KCl, 1 mM EDTA, 1 mM EGTA, 0.1% BSA). For MOMP assays, mitochondria were incubated in TMIB supplemented to 110 mM KCl (trehalose mitochondrial assay buffer, TMAB), ±caspase-8 cleaved mouse BID (R&D Systems) or peptides (final concentrations are indicated in the text and figure legends) for 60 minutes at 37°C. Reactions were then fractionated into supernatant and pellet by centrifugation at 5,500×g for 5 minutes and analyzed by SDS-PAGE and western blot with anti-cytochrome *c*. Supernatant and pellet fractions were separated using the Criterion XT 4–12% gel system (Bio-Rad) with 1X MOPS buffer at 150 V. Proteins were transferred to nitrocellulose by standard western conditions, blocked in 5% milk/TBST and the primary antibody (in blocking buffer: cytochrome *c* 1∶2000, clone 7H8.2C12 Pharmingen) was incubated overnight at 4°C. The secondary antibody (1∶5000 in blocking buffer) was incubated at 25°C for 1 hour before standard enhanced chemiluminescence detection. For MOMP assays, all peptides were resuspended in anhydrous DMSO (5 mM stock) in a N_2_ environment, aliquoted, stored at −80°C and thawed only once. To obtain BAK- and BAX-deficient mitochondria, heavy membrane fractions were isolated from the livers of polydIdC-treated *MxCre bak ^−/−^ bax ^f/−^* mice. Where indicated, total cytochrome *c* was determined by a sample containing mitochondria solubilized in 0.5% CHAPS.

### Infectious animal model

Six- to eight week old female Balb/c mice (Jackson Laboratory, Bar Harbor, ME) were maintained in a Biosafety Level 2 facility in the Animal Resource Center at SJCRH. All experimental procedures were done under general anesthesia with inhaled isoflurane 2.5% (Baxter Healthcare Corporation, Deerfield, IL). Infectious agents and peptides were diluted in sterile PBS and administered intranasally in a volume of 100uL (50uL per nare) to anesthetized mice held in an upright position. Groups of six to ten mice were weighed and followed at least daily for illness and mortality. The infectious dose for all experiments was 100 TCID_50_, which with these stocks caused 10–15% weight loss and no mortality.

### Ethics statement

All experimental procedures were approved by the Animal Care and Use Committee at SJCRH under relevant institutional and American Veterinary Medical Association guidelines and were performed in a Biosafety level 2 facility that is accredited by AALAAS.

### BAL for cellular cssessment

Following euthanasia by CO_2_ inhalation, the trachea was exposed and cannulated with a 21 gauge plastic catheter (BD Insyte, Becton Dickinson, Sandy, UT). Lungs were lavaged thrice with 1mL of cold, sterile PBS. Flow cytometry (FACs Calibur, Becton Dickinson, San Jose, CA) was performed and the number of white blood cells (WBC) per mL determined (Hemavet 3700, Drew Scientific, Dallas, TX) on the BALF suspension after red blood cell depletion using Red Cell Lysis Solution (Sigma). Briefly cells were stained with 1uL/10^6^cells Gr1(FITC)/F480(PER-CP) for 20 min, washed then resuspended in 100uL FACS wash buffer. The proportions of Neutrophils (Gr1^+^ within the cellular region), Macrophages (F480^+^ within the cellular region) were assessed as a proportion of cellular events analyzed by flow cytometry as related to the number of WBC/mL.

### Histopathologic examination

Lungs were removed immediately following euthanasia via CO_2_ inhalation, sufflated and fixed in 10% neutral buffered formalin overnight. The lungs were processed routinely, embedded in paraffin and sectioned at 5um. For histopathologic examination, slides were stained with hematoxylin and eosin and examined microscopically as previously described [10]. Production of myeloperoxidase (MPO) by affected tissue was detected via immunohistochemical staining by the Veterinary Pathology Core Laboratory at SJCRH. This was done on a LabVision autostainer at room temperature using tris-buffered saline rinses between steps. Endogenous peroxidase activity was blocked by incubation with 3% H_2_O_2_ (Humco, Texarkana, TX) for 5 minutes. Slides were incubated with rabbit anti-human myeloperoxidase (DAKO, Carpinteria, CA, cat # A0398) at 1∶1500 for 30 minutes followed by incubation with horse-radish peroxidase-conjugated Rabbit-on-Rodent Polymer (BioCare Medical, Concord, CA, cat # RMR622) for 30 minutes. Slides were incubated for 5 minutes with the chromagen DAB (3,3′ diaminobenzidine tetrahydrochloride, cat # K3466, DAKO). Hematoxylin (ThermoShandon, TA-125-MH) was used as counterstain. Grading and description of pathology were performed by an experienced veterinary pathologist (KLB) blinded to the composition of the groups using methods previously described [33].

### Statistical analysis

Comparison of cell death and cellularity of BAL fluid between groups was done using analysis of variance (ANOVA) for multiple comparisons and the Student's t-test for matched, single comparisons. A p-value of <0.05 was considered significant for these comparisons. SigmaStat for Windows (SysStat Software, Inc., V 3.11) was utilized for all statistical analyses.

## Supporting Information

Figure S1 Uptake of PB1-F2 derived peptides into cells. J774 macrophages were incubated with either A) N-terminal PB1-F2 peptide:streptavidin:Cy2, B) streptavidin:Cy2, or C) C-terminal PB1-F2:streptavidin:Cy2 for 5 hours. Representative sections are pictured. D) Confocal fluorescent images were taken every 20 minutes from 6 specific locations for 16 hours. Representative cells showing uptake of PB1-F2 peptide, cytoplasmic distribution, and degradation are shown at the timepoints pictured. This compares favorably to distribution and kinetics of the full length PB1-F2 when expressed from virus [15], although peptide concentration peaks a few hours earlier since viral replication is not required. Green staining represents PB1-F2, blue is nuclear staining, and red is cellular membrane.
(3.60 MB TIF)

Figure S2 Expression of PB1-F2 in infected cells. MDCK cells were infected with a panel of viruses or were mock infected, and expression and localization of PB1-F2 was examined by confocal microscopy 12 hours later. No expression of PB1-F2 was detected in this assay for viruses engineered so that the ORF for the PB1-F2 protein was disrupted.
(2.71 MB TIF)

Text S1 Supplementary methods. Confocal laser scanning microscopy and immunofluorescence.
(0.03 MB DOC)
