# Supplementary figures and images for: PB1-F2 Proteins from H5N1 and 20th Century Pandemic Influenza Viruses Cause Immunopathology
Source: PLoS Pathog. 2010 Jul 22;6(7):e1001014. doi: 10.1371/journal.ppat.1001014 (PMC2908617; doi:10.1371/journal.ppat.1001014)

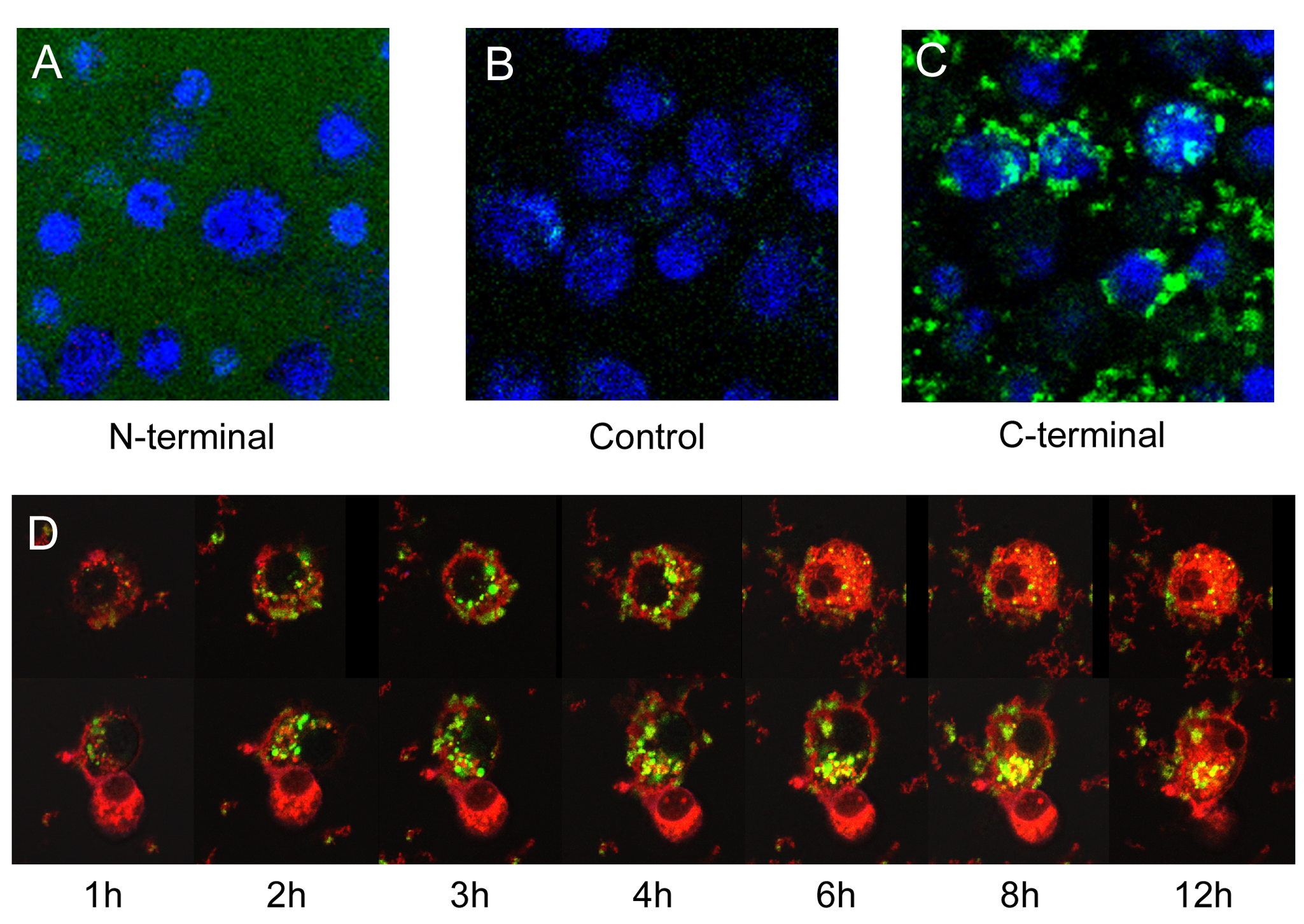

Supplement: Figure S1 — Uptake of PB1-F2 derived peptides into cells. J774 macrophages were incubated with either A) N-terminal PB1-F2 peptide:streptavidin:Cy2, B) streptavidin:Cy2, or C) C-terminal PB1-F2:streptavidin:Cy2 for 5 hours. Representative sections are pictured. D) Confocal fluorescent images were taken every 20 minutes from 6 specific locations for 16 hours. Representative cells showing uptake of PB1-F2 peptide, cytoplasmic distribution, and degradation are shown at the timepoints pictured. This compares favorably to distribution and kinetics of the full length PB1-F2 when expressed from virus [15], although peptide concentration peaks a few hours earlier since viral replication is not required. Green staining represents PB1-F2, blue is nuclear staining, and red is cellular membrane. (3.60 MB TIF) [file ppat.1001014.s001.tif]

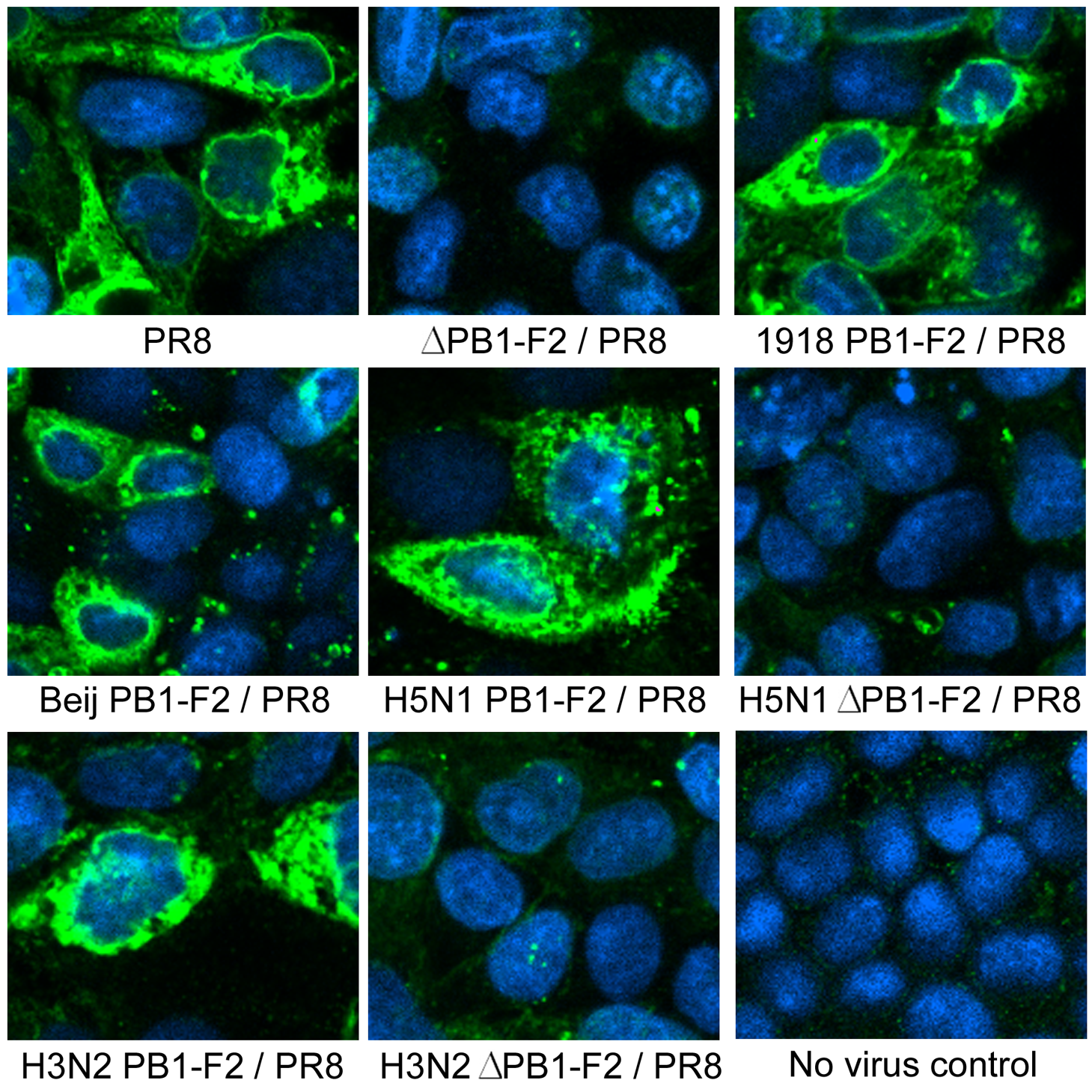

Supplement: Figure S2 — Expression of PB1-F2 in infected cells. MDCK cells were infected with a panel of viruses or were mock infected, and expression and localization of PB1-F2 was examined by confocal microscopy 12 hours later. No expression of PB1-F2 was detected in this assay for viruses engineered so that the ORF for the PB1-F2 protein was disrupted. (2.71 MB TIF) [file ppat.1001014.s002.tif]
