# Supplementary material for: PB1-F2 Proteins from H5N1 and 20th Century Pandemic Influenza Viruses Cause Immunopathology
Source: PLoS Pathog. 2010 Jul 22;6(7):e1001014. doi: 10.1371/journal.ppat.1001014 (PMC2908617; doi:10.1371/journal.ppat.1001014)
Supplement: Text S1 — Supplementary methods. Confocal laser scanning microscopy and immunofluorescence. (0.03 MB DOC) [file ppat.1001014.s003.doc]

**Text S1. Supplementary Methods**

**Confocal laser scanning microscopy and immunofluorescence.** To examine the kinetics of uptake of PB1-F2 derived peptides into cells, J774 macrophages were plated out at 1 x 10^6^ cells per well on a 4 well chambered coverglass (Labtek) and incubated at 37° overnight. Cells were washed with sterile J774 infection media prior to the addition of 27-mer peptides derived from the PR8 PB1-F2 protein or control. The fluorophore cyanin (Cy2) was conjugated to streptavidin and a biotinylated version of the C-terminal PR8 peptide highlighted in Figure 1. A 27-mer peptide derived from the N-terminal region of the PR8 PB1-F2 peptide and streptavidin:Cy2 without peptide were used as controls. The cells were placed on a fluorescent microscope (Nikon C1Si lens on a TE2000 microscope) and time points taken every 20 minutes for 16 hours as described [15]. In some experiments Texas red-coupled wheat germ agglutinin (WGA) was included as a non-specific membrane stain to facilitate visualization of the same cells over time.

To demonstrate that full length PB1-F2 protein is not expressed from viral constructs where the PB1-F2 reading frame has been altered to abrogate such expression, MDCK cells were grown on glass coverslips and infected with viruses as described in detail previously [15]. Briefly, 12 hours after infection, cells were fixed with 4% formaldehyde, permeabilized, washed, and incubated with a primary antibody directed against the N-terminal region of PB1-F2 (gift of Jon Yewdell, NIH) as described [15]. After washing cells were then incubated with a secondary antibody (Alexa-Fluor 488-conjugated donkey anti-rabbit antibody) and mounted with DAPI for nuclear staining [15]. Cells were visualized and pictures taken with a Nikon ClSi lens on a TE2000 microscope.
